# Why Map Issues? On Controversy Analysis as a Digital Method

**DOI:** 10.1177/0162243915574602

**Published:** 2015-09

**Authors:** Noortje Marres

**Affiliations:** 1 Goldsmiths, University of London, London, United Kingdom

**Keywords:** controversy analysis, media technologies, digital methods, methodology, media bias, politics of knowledge, politics of technology, Twitter, Internet governance

## Abstract

This article takes stock of recent efforts to implement controversy analysis as a digital method in the study of science, technology, and society (STS) and beyond and outlines a distinctive approach to address the problem of digital bias. Digital media technologies exert significant influence on the enactment of controversy in online settings, and this risks undermining the substantive focus of controversy analysis conducted by digital means. To address this problem, I propose a shift in thematic focus from controversy analysis to issue mapping. The article begins by distinguishing between three broad frameworks that currently guide the development of controversy analysis as a digital method, namely, demarcationist, discursive, and empiricist. Each has been adopted in STS, but only the last one offers a digital “move beyond impartiality.” I demonstrate this approach by analyzing issues of Internet governance with the aid of the social media platform Twitter.

Media are never impartial, they always participate.

David Garcia and Geert Lovink (1998)

## Introduction

Digital media technologies are ubiquitous, but there continue to be widespread concerns about the “bias” of online information and knowledge. Commentators still sound the alarm about the dangers inherent in the spread of dubious claims via digital media, as when the well-known Internet critic Evgeny Morozov cried foul of “dodgy” anti-vaccine activists, who have “half a million followers on Twitter.” In a popular online article, he argued that it was time to build proactive measures into Internet infrastructures, most notably by having search engines identify and label suspect sources as “compromised.”^1^ Morozov’s red banner proposal itself sets alarms ringing and was probably designed for that purpose. In setting up the search engine as arbiter, Morozov’s proposal effectively places these powerful digital platforms beyond the reach of “bias critique.” As a central institution of the digital information economy, however, search engines have been criticized for introducing bias into online environments, most notably via their selection and ranking algorithms. These tend to favor popular, fresh, and institutionally accredited sources (Introna and Nissenbaum 2000; Gillespie 2013).

Persistent public concern with bias in the digital context poses several challenges for the study of science, technology, and society (STS), and recent work in STS has certainly found ways to engage with the situation. STS researchers have used the “scandal” of the biased nature of digital information to make the case, once again, for a less negative, more generous understanding of the politics of knowledge (Latour 2011; Rogers and Marres 2000). Specifically, they have proposed that digitization makes possible the further development of controversy analysis, a distinctive approach for studying the partiality of knowledge (see also Leydesdorff and Hellsten 2006; Venturini 2012). It was through historical and fieldwork studies of controversies about scientific issues that STS had established its distinctive claim that the formulation of knowledge claims and the organization of political interests tend to go hand in hand (Bloor 1982; Collins and Pinch 1998; Hagendijk and Meeus 1993). In the early 2000s, this methodology was used to analyze the politics of *digital* knowledge and information (Rogers and Marres 2000; Prabowo et al. 2008). For more than a decade, efforts have been underway to render STS methods of controversy analysis compatible with the new sources of data and analytic techniques spawned by the Internet and wider processes of digitization. As I will discuss below, this has resulted in various implementations of controversy analysis as a digital method, but the project continues to face significant problems, including the problem of digital bias.

Digital methods of controversy analysis are potentially biased because the instruments they deploy to describe controversy—search engines and social media platforms—exert a notable influence on the enactment of controversy online (Madsen 2012), which places serious limits on the generalizability of the insights of digital controversy analysis. Digital bias threatens to undermine controversy analysis because we cannot be sure that we are analyzing the controversies themselves, rather than the digital settings that render these controversies analyzable (Venturini and Guido 2012). STS-informed work in digital controversy analysis has proposed various ways of addressing this challenge. Drawing on insights from the Strong Programme into the inherent partiality of knowledge (Barnes, Bloor, and Henry 1996), STS-informed analyses of digital controversies *expect* the organization of content and the mobilization of interests to go hand in hand in digital settings. In this article, I take up this “affirmative” approach to bias in the digital analysis of controversies, showing how it can be developed into a viable empirical strategy. I argue that if we are serious about affirming the “influence of the setting” in the enactment of controversy online, then we must adopt a more open-ended approach and not just analyze controversies but map issues.

## Situating Controversy Analysis as a Digital Method

Broadly defined, controversy analysis as a digital method involves the use of computational techniques to detect, analyze, and visualize public contestation over topical affairs (for a discussion, see Marres and Rogers 2005). Importantly, while methods of controversy analysis have been central to the development of STS over the last decades, the digital implementation of controversy analysis is best understood as *an interdisciplinary undertaking*. Different fields currently contribute to this project including the sociology of science and technology, computer science, media studies, communication, and policy analysis (Thelwall, Vann, and Fairclough 2006; Benkler 2012; Chateauraynaud 2009; Rogers and Marres 2000; Rogers and Ben-David 2008; Yasseri et al. 2012; Venturini 2012) as well as various professional fields including design, journalism, and advocacy (Marres and Weltevrede 2013; Borra et al. 2014).^2^ Although there are notable differences between approaches, work across these fields deploys digital techniques for the capture, analysis, and visualization of—often Internet-based—data in order to render legible disputes about public issues. Building on existing approaches developed in the above fields from the 1970s to analyze public and policy debates and enable intervention in these debates, analysis of digital controversies has clear affinities with the applied research method of “debate mapping” (for a discussion see Rogers 2009; Whatmore 2009).

The rise to prominence of the web from the mid-1990s onward offered significant new opportunities for the implementation and development of controversy analysis (Rogers and Marres 2000; Latour 1998; Thelwall, Vann, and Fairclough 2006). It is not just that the digitization of social life has made available *masses of data* that are useful for the study of controversy. Digital sources also tend to be *organized or structured* in ways that make them highly suitable for controversy analysis to trace the unfolding of disputes across different sites as well as through time (Venturini 2012; Marres and Rogers 2005). Third, the digital data explosion has been accompanied by a proliferation of digital *instruments for data analysis and visualization*, many of which are suitable for controversy mapping, such as network and textual analysis and visualization. These prominently include web-based tools, which can be accessed online in order to locate, analyze, and visualize networks of sources, more or less in real time (Rieder 2013).

**Figure 1.**
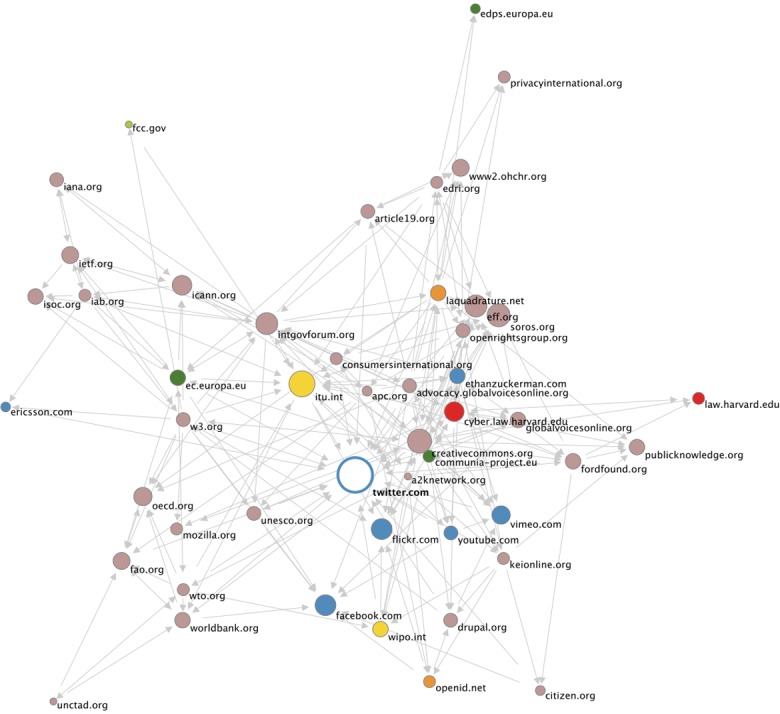
World Conference on International Telecommunications (WCIT) network on the web located with the aid of IssueCrawler, December 2012.

For example, Figure 1 shows a so-called issue network located on the web with the aid of hyperlink analysis. This network was found with the aid of *IssueCrawler*, a web-based tool that delineates topical formations online by crawling, analyzing, and visualizing hyperlinks on the web. This particular network brings together sources dealing with the World Conference on International Telecommunications (WCIT) that took place in Dubai in December 2012, which became the focus of debates about Internet governance during this time. What distinguishes this formation from other types of online networks is its “issue specificity”: the sources it brings together each address a current affair, in this case, WCIT. Importantly, such a topical assemblage is delineated only by following and analyzing hyperlinks from starting points (web pages) suggested by users as relevant to the issue at hand—in the case of Figure 1, by two experts on issues of Internet governance. The *formal* technique of crawling and analyzing hyperlinks then provides a way to locate *substantive* formations online, making these networks available for further examination, for instance with the aid of textual analysis (Marres and Rogers 2000, 2005; see also Leydesdorff and Hellsten 2006).

Digital techniques for network and textual analysis thus offer potentially powerful instruments for controversy analysis. To be clear, these techniques are used for a variety of purposes including trend mapping and social network analysis (Mutzel 2009), but they nevertheless make a good match for the methodological sensibilities of controversy analysis by allowing us to analyze public disputes across “heterogeneous” domains, such as science and the media, or governmental and civil society sources. In this spirit, a younger generation of researchers has taken up digital tools of network and textual analysis to map controversies online, including climate change (Venturini and Guido 2012; Marres and Rogers 2000; Niederer 2013), food technologies (Beck and Kropp 2011; Marres and Rogers 2000), biofuels (Eklof and Mager 2013), nanotechnology (Madsen 2013), and the Fukishima disaster (Plantin 2011; Moats 2014). Although these studies have attracted significant interest, it is not always self-evident what methodological innovation precisely they undertake, as computational techniques of network and textual analysis have been used since at least the 1980s to detect dynamics of controversy in electronic databases of scientific journal articles (Callon, Law, and Rip 1986; Leydesdorff and Hellsten 2006). Indeed, the availability of digital analytic techniques or digital networked data, in and of itself, cannot explain what is new or specific about current efforts to implement controversy analysis by digital means. Rather, it has to do the manner in which the wider apparatus of controversy analysis is being configured (Marres 2012).

Relevant here is that controversy analysis is deployed interactively online as a way to *intervene* in networked information environments, and that interactive applications have been put forward in recent years for the online analysis of knowledge disputes with the explicit aim of mitigating against the bias of online content. Morozov’s provocative proposal was inspired by a prototype application developed by Intel Research called “dispute finder,” which provides web users with an overview of contesting claims whenever they browse a disputed information source (Ennals et al. 2010).^3^ Insofar as digital methods of controversy analysis are deployed not just to analyze but to interactively intervene in online information environments, they can be called *interested methods* (Asdal 2014). They present a site where the apparatus for the evaluation of online information is currently being assembled, and in this undertaking not just epistemic but also political and economic normativities come into play. To better understand what is at stake in the configuration of controversy analysis as a digital method and how STS can intervene in relation to this broader endeavor, I distinguish between three different frameworks that give direction to this project.

## Three Frameworks for Digital Controversy Analysis: Demarcation, Discourse Analysis, and Radical Empiricism

*Demarcationists* make the strongest case for the digital implementation of controversy analysis. Reflecting public concern about the biased nature of networked information, demarcationists aim to deploy computational methods of controversy analysis to delineate legitimate from illegitimate knowledge sources and disputes. The dispute finder prototype presents an applied example, but the approach also informs projects in large-scale data analysis, such as the study of controversy on the online encyclopedia platform Wikipedia undertaken by Yasseri and colleagues (2012; for another example, see Weber, Garimella, and Borra 2012). Analyzing a sizable set of Wikipedia articles using statistical methods, this project developed a technique for detecting the “controversiality” of topics on Wikipedia. Proposing indicators like the number of edits, and “mutual edits” (reverts), to establish the relative level of “substantive disagreement” in Wikipedia articles, the project produced rankings of the most controversial Wikipedia topics, including a “top 10” which was recently featured in *The Economist* magazine (“global warming” made it into the English-language top 5 and “Sigmund Freud” into the French one; “Daily Chart, Edit Wars” 2013). The project also formalized a procedure for identifying sites of epistemic contestation, which Yasseri et al. (2012) define as conflicts with an “internal” cause (as differentiated from disputes caused by “external events” and thus not concerned with knowledge claims!). Adopting an “internalist” understanding of knowledge controversies, this work seeks to implement the prescriptive ambition of twentieth-century philosophy of science to demarcate legitimate or relevant knowledge disputes from illegitimate or irrelevant disagreements about nonepistemic things.^4^

The *Discursive* approach to digital controversy analysis builds on sociological methods of discourse analysis, for which the objective is not to determine the status of statements or topics as such but to map positions in a debate (Beck and Kropp 2011; Yaneva 2012; Venturini, Gemenne, and Severo 2013). Here, controversy analysis serves exploratory purposes, namely, to detect relations between substantive arguments and socially and politically located actors and to render such relations available for interpretation by various audiences (Beck and Kropp 2011). In many cases, researchers do this by analyzing which claims and issue terms have support from which actors, demonstrating which issues are becoming subject to contestation between heterogeneous actors. Thus, Beck and Kropp (2011) produced detailed discursive maps of food security debates, showing how the controversy over the food-coloring agent “beta-carotene” in Germany in the early 2000s brought food producers, retailers, and consumers into relations with one another.^5^ The objective is to detect socio-epistemological formations and to render these patterns visible for academic, professional, and ideally, lay audiences. Such a discursive approach to controversy analysis is adopted by many social scientific projects in controversy mapping, including those informed by STS (Beck and Kropp 2011; Eklof and Mager 2013; Leydesdorff and Hellsten 2006).

The discursive project to map substantive statements (“knowledge content”) onto social interests resonates well with STS sensibilities and evokes the principle of the Strong Programme that *all knowledge content* is are likely to be associated with factional interests of a more or less determinate kind. However, STS-informed projects of digital controversy analysis have also attempted to move beyond it. Significantly, these efforts reflect the influence of actor–network theory (ANT).^6^ Bruno Latour and colleagues developed a range of software tools and research protocols that facilitate the digital implementation of ANT, and controversy analysis has provided the overarching framework for much of this work (Venturini 2012; Latour 1998; see also Yaneva 2012; Munk 2013). Richard Rogers, colleagues, and I have drawn on ANT in the development of digital methods of issue mapping in the development of the IssueCrawler, the web-based tool for the analysis of “issue networks” on the web presented in Figure 1. These various ANT-informed approaches are similar to the discursive perspective, but they also make distinctive assumptions that expand and complicate it.

Crucial in this respect are the empirical capacities of controversy analysis. One way in which recent work in STS has built on the Strong Programme is by *extending* the empirical scope of controversy analysis. Controversies, according to this work, do not just bring into view relations between scientific statements and social or political interests, they also provide an “empirical occasion” for a wider social inquiry, that is, controversies render visible relations between science, technology and society, making these available for analysis (Collins and Pinch 1998; Latour 2005). In what I call the *empiricist implementation of controversy analysis* as a digital method, this ambition is extended to online settings. This approach proposes that controversy in digital media settings presents us with especially useful or productive empirical occasions, that is, they can tell us what the issues of contestation are, who the actors are, and where they are based (Marres and Rogers 2009). However, while STS scholars previously turned to controversies in order to analyze relations between actors, non-human entities, institutions, practices, and so on digital researchers today have taken up the approach to practice *controversy detection.* With the aid of digital methods like the issue-network visualization shown in Figure 1, we can determine *whether* a given topic constitutes a controversial issue: did an active network organize online around a topic like WCIT? If so, do the pages in the network engage in contestation and, if so, what about?^7^

Different analytic frameworks then guide the digital implementation of controversy analysis. To be sure, demarcationists, discursivists, and empiricists share various assumptions. Both demarcationists and empiricists are interested in the detection of controversy dynamics, using techniques to determine what are relevant, active topics of controversy. Both discursivists and empiricists analyze the *composition* of controversies: who are the actors? where are they based? what is relevant issue language? how do they change over time? But there are also significant differences. Although demarcationists deploy controversy analysis to *adjudicate* between sources, discursivists’ primary aim is to facilitate the *exploration* of controversy. Demarcationists propose that knowledge controversies should be clearly distinguished from nonepistemic debates online, whereas discursivists and empiricists deploy digital methods in order to demonstrate the entangling of epistemic and political dynamics. Finally, discursivists posit a social ontology of controversy stipulating actors, positions, and societal domains. Empiricists, however, seek to minimize ontological assumptions, arguing that controversy in digital settings is heterogeneously composed in ways that can’t, and shouldn’t, be predetermined by the analyst. Instead, they ask are the issues enacted through policy reports or in situ protests? Communicated through pdfs or tweets?

I believe that discursive and empiricist approaches are the best suited to pursue the intellectual and normative project invoked in the introduction, “to move beyond impartiality” in the analysis of knowledge, technology, and society—to develop an understanding of the biases of digital information in a way that does not fall back on the imagined ideal of neutral, noninterested, knowledge (Venturini 2012). However, considering the perceived societal relevance—and computational implementability—of demarcationist approaches to controversy analysis, it is crucial that we offer a clear definition of the latter project. In a context in which “digital bias” is widely perceived as a public problem, what do we gain by “moving beyond” the ideal of the impartiability of knowledge? I argue that this long-standing project faces important new challenges in digital environments, as problems of bias there pertain not only to content but also to the settings of controversy. This, in turn, has methodological implications for what is required to “move beyond impartiality” in digital research. I argue that the empiricist approach is especially well equipped to satisfy these requirements.

## Two Approaches to Problems of Digital Bias in Controversy Analysis

Online environments pose significant problems for the implementation of controversy analysis, and digital bias is one of them. Each of the frameworks introduced above recognizes that digital media technologies can*not* be considered neutral. Some STS-informed studies of online controversies are specifically concerned with the problem of digital bias, demonstrating how online devices like search engines and platforms like Wikipedia exert significant influence on the mediation of controversies online.^8^ Eklöf and Mager (2013) have compared the presentation of controversial “biofuels” in the press and in search engines, showing that the latter are more biased toward commercial sources (see also Madsen 2013), and others have demonstrated the biases in Wikipedia reporting on specific issues like climate change and nuclear energy toward industry and scientific sources (Niederer 2013; Weltevrede and Borra 2013, Moats 2014). Of course, STS scholars have for many decades been interested in media bias and the influence it exerts on what claims and actors gain public attention during controversy (Nelkin 1979; Hilgartner 2000). In digital controversy analysis, however, the question of bias touches on a deep methodological problem concerning the viability of digital media as settings for the enactment of controversy, and their analysis.

This problem is framed and addressed in very different ways by the different frameworks introduced above. Discursivists frame digital bias in negative terms, treating it as a source of noise that might undermine the epistemic credibility of digital controversy analysis because online information is partial and biased, a controversy analysis that relies primarily on this type of information will suffer from the very same problem (Venturini and Guido 2012). For this reason, discursivists tend to advocate the use of data from mixed sources (both online and off-line), arguing that controversy analysis must take active steps to militate against online biases by “purging” analysis of their effects. In this vein, Thelwall, Vann, and Fairclough (2006) recommend that in conducting issue analysis with the web, it is advisable to “remove from the data wherever possible all occurrence of web phenomena that serve to obscure [the issue]” (see also Rogers 2013). Whenever the process of online data capture results in some sources figuring more prominently than others in the data set (e.g., because some sources receive comparatively more hyperlinks than others), this effect has to be neutralized by removing duplicates (see also Pearce et al. 2014).

Others, however, have questioned the suitability of this “precautionary” approach. Advancing an “affirmative” approach to digital bias, they propose that the online dynamics that precautionists define negatively as sources of noise or corruption of data may also present a positive, constitutive aspect of controversy online (Marres and Rogers 2009). The use of hyperlink analysis for controversy research helps to make this clear. On the one hand, hyperlinking presents a socio-technical phenomenon that is specific to digital networked media, and accordingly hyperlink analysis can be used to demonstrate biases that are specific to these settings. We can ask, for instance, whether overall hyperlink patterns are relatively centralized or de-centralized (Kelly 2010) or whether and how innovations in hyperlinking, such as the introduction of Twitter or Facebook buttons, influence which type of sources feature prominently online (Gerlitz and Helmond 2013). However, hyperlink analysis may also be used to detect substantive dynamics of controversy online, as in the case of the issue-network presented in Figure 1. Digital devices like hyperlinks may introduce effects of digital bias into online content, and as such are reflective of media technological dynamics. But as they provide instruments for the organization of issues online, they may equally carry a substantive “charge.”

The affirmative approach to digital bias acknowledges and exploits the ambiguity of digital devices, arguing that we can rely on them as empirical means for detecting controversy dynamics (Marres and Rogers 2005). One of the striking features of digital settings like the web is the close connection between technological dynamics and dynamics of topic or issue formation (see also Schneider and Foot 2005), and it is often unclear which of these two dynamics we are dealing with when analyzing controversies online. To return to the example of the WCIT issue-network presented in Figure 1, the fact that the social media platform Twitter is the central node in this network could be due to a variety of effects: it could be because Twitter buttons and feeds have become increasingly common on the web, or because Twitter presents a key site of mobilization in the controversy around the WCIT. That hyperlink analysis suggests Twitter as a relevant source may then be due either to media technological dynamics of “digital bias” or to the substantive dynamics of the controversy, or both.

So there are two very different ways to treat the methodological problem of digital bias in online controversy analysis, that is, the precautionary approach treats digital media technologies as a source of noise that must be neutralized, while the affirmative approach treats digital devices as an empirical resource for controversy analysis. The former proposes that digital content must be disembedded from online settings in order to secure the validity of issue analysis. The latter seeks to bring publicity devices that are specific to digital culture within the empirical frame of controversy analysis.^9^ To be clear, both approaches recognize that digital devices like hyperlinks may result in the privileging of some sources over others in online settings. Hyperlinks do not offer “neutral” tools for delineating data sets, they are instruments for the organization of networked information, and as such they participate in the (de-)valuation of digital content. Where the two approaches differ is on the methodological question of whether controversy analysis must militate against these effects, or rather affirm their role in the enactment of controversy online.^10^ The affirmative approach proposes that digital devices are in part *formative and therefore potentially* *indicative* of controversy dynamics online. They organize sources in ways that bring substantive contestations to the fore (Gillespie 2013).

The three frameworks introduced above are associated with one of the two approaches to digital bias. Discursivists tend to adopt a precautionary stance, as their aim is to map “positions in a debate.” Indeed, the metaphor of “debate” is generally deployed to dis-embed contributions from media technological settings (Thompson 2011). As we have seen, empiricists are inclined to defer to fieldwork settings to answer empirical and sometimes even ontological questions, and accordingly they are generally quite happy to rely on technical formations like a hyperlink network to tell them who the actors and what the issues are. Demarcationists might go either way. While a focus on substantive disagreement tends to go with a negative understanding of technological bias, this is not always the case. Yasseri et al. (2012)’s project on Wikipedia controversies leans toward an affirmative approach to digital bias, as it relies on the measurements of platform-specific features such as the number of page edits to determine the “controversiality” of Wikipedia articles. In this sense, one’s approach to digital bias is *not* predetermined by the broader normative framework for controversy analysis. However, the affirmative approach to digital bias is in my view of critical importance for the further development of controversy analysis as a digital method. It provides a way to translate the project of the move beyond partiality in the social study of knowledge, technology, and society into a methodological strategy for digital research. In the next sections, I discuss how this is so, but first I want to consider a key problem with the affirmative approach.

## The Promise and Problem of an Affirmative Approach to Online Bias

The proposal to affirm media bias in the empirical study of controversy is certainly not a new proposal. Especially useful in this regard is Hilgartner’s (2000; drawing on Bogen and Lynch 1989) discussion of the problem of the “warm record” in controversy analysis. Hilgartner argues that media accounts of controversial affairs can under no circumstances be treated as neutral records of controversy, *because the act of publicizing a controversy*—for instance, by sending out a press release or leaking policy documents to the press—*inevitably* *constitutes an intervention in controversy*. In other words, public records of controversy are not external to the controversy but partly internal to and inflected by it. An affirmative approach to the bias of media technologies can also be recognized in scientometrics, a well-established analytic approach that relies on citations and other formal features of scientific journal articles—such as the key words used to index articles—to investigate the dynamics of scientific fields (Leydesdorff 2001). As it analyzes and visualizes citation and key word relations, scientometrics also deploys formal devices that are specific to a publicity genre—the scientific journal article—in order to address substantive questions: Who are the principal actors? Which topics are prominent in this field?^11^

Indeed, digital methods of controversy analysis have been defined as the attempt to extend scientometric methods to new media environments (Scharnhorst and Wouters 2006). And it can be argued that the digital equivalents of publication, citation, and indexation allow not just for the extension but the *expansion* of the analytic capacities of network and textual analysis as compared to their predigital counterparts. Whereas citation analysis used to be limited to the scientific literature, digital devices like hyperlinks and hashtags are deployed across domains, from science to advocacy, journalism, policy, and activism, allowing us to study the interrelations between fields. Second, the rise of digital platforms for user-generated content—“social media”—has broadened the range of digital devices available as empirical resources for controversy analysis. Besides linking, online platforms such as Twitter and Facebook enable several other “informational actions” such as “tagging,” “following,” “sharing,” and “mentioning” (Rieder 2013). To be sure, the rise to prominence of such “information actional” formats presents important topics for the social study of media technology in their own right (Crawford and Gillespie 2014). But they also present promising instruments for controversy analysis, perhaps most of all hashtags, the key words identified and applied by users as tags to identify relevant topics in social media content.

**Figure 2.**
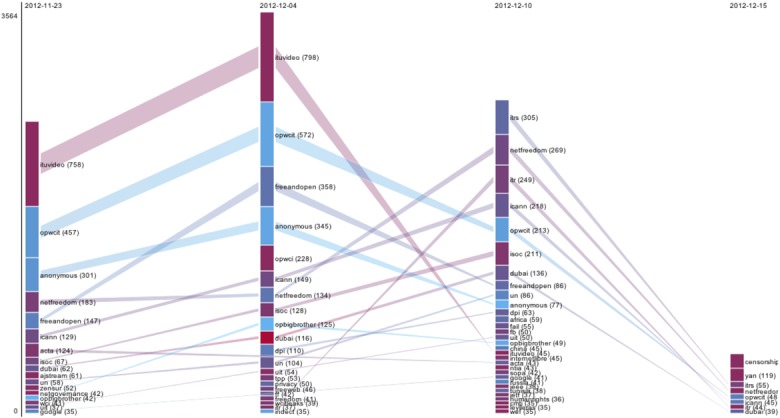
Hashtag profile for “World Conference on International Telecommunications (WCIT),” showing its hashtags associations per interval (before, during (×2), and after the summit), produced with the associational profiler, February 2013.

Like the key words used to index scientific articles, hashtags can be analyzed to detect emerging topics. When faced with a relatively opaque and complex topic such as the WCIT, issue detection becomes especially important (Hofmann, 2013), and hashtag analysis offers a useful instrument for this. Thus, in our WCIT case study, we analyzed the hashtags used on Twitter in relation to this topic in the period surrounding the summit, in order to determine to which issues WCIT is related, and how “active” these are (see Figure 2).^12^ As it turned out, the profile of the WCIT hashtag on Twitter contained a high proportion of campaign and issue terms (surveillance, bigbrother, and privacy), and this may be taken as a rough indication of the controversiality of WCIT. However, our hashtag analysis also points toward some problems with our reliance on tags for analyzing controversy. This problem can be summed up in the question, are we mapping controversies or the effects of media technology? Above I suggested that the composition of an “issue network” located with the aid of hyperlink analysis may be indicative of either substantive or media technological dynamics. Something similar applies to hashtags on Twitter. When we analyze hashtag relations, are we analyzing which type of messages are more likely to be accompanied by hashtags? Are we mapping the privacy settings of different sources, where some remain inaccessible to our online data tools?

The problem is tenacious in the analysis of social media data, as these platforms are explicitly designed to facilitate promotional forms of publicity (i.e., advertising). Hashtags are one of the principal instruments for gaining an audience in these settings and are widely used to that effect by marketers in ways that frequently have little to do with the informational content being “pushed” (Gillespie 2010). Indeed, our WCIT hashtag analysis showed not only that WCIT is associated with issue terms such as “Internet freedom” (#netfreedom), deep packet inspection (#dpi), and censorship but also that equally prominent in relation to WCIT on Twitter were more seemingly generic tags like #anonymous, referring to the anonymous “hacktivist” collective, which has a reputation for latching onto any content with “currency” to gain attention (Coleman 2011). While we set out to map a controversy in online media, we may easily end up analyzing phenomena that tell us more about digital media platforms and practices than about the controversy in question. To affirm the bias of online settings in digital controversy analysis does not simply enhance the empirical capacities of controversy analysis, it comes at significant price, that is, it puts at risk the substantive focus of digital controversy analysis.

In order for an affirmative approach to digital bias to be methodologically viable, we must find ways to ensure that we map *controversy dynamics*, rather than *media technological dynamics*. Digital bias is a problem for controversy analysis, but the problem I flag here is different from the one highlighted by precautionists who do not really recognize that acts of publicity—interventions that push certain topics, actors, and locations into the foreground—are part of the empirical object of controversy analysis (since they propose that we should actively disregard such publicity effects and remove this bias from the data). A different problem of digital bias comes into focus once we recognize publicity effects are in part constitutive of controversy: the problem of the *inherent ambiguity* of the empirical object of online research. The recognition that instruments of digital publicity like hyperlinks and mentions may help to produce controversy does not relieve us from the obligation to configure a robust empirical object.^13^ In the remainder of this article, I would like to discuss ways to address this challenge. I argue that if we affirm the participation of digital media technologies in controversy, then we must redefine the empirical object of controversy analysis, that is, we must map issues and not only controversies.

## From Controversy Analysis to Issue Mapping

Adopting an affirmative approach to digital bias is a methodological choice, but it also raises empirical questions, that is, how are digital media technologies affecting the manner in which controversies are conducted in our societies? If we affirm that digital media technologies participate in the enactment of controversy online, then surely digital controversy analysts must take a positive interest in how they inflect public controversy and the forms it takes today. The online controversy around the WCIT again provides a useful example: one significant intervention in this controversy took the form of a digital act of publicity, namely an “information leak.” While the conference was still going on, a large number of official summit documents, which had not previously been made public, were made available for download via websites like dot-nxt.com (Personal communication, anonymous source). On the one hand, such a “data dump” is a form of publicity that is to an extent specific to Internet culture (Coleman 2013; on leaks as an intervention in controversy, see also Hilgartner 2000). At the same time, however, this intervention can be understood as contextually specific to the WCIT controversy. Unlike other recent Internet-related international summits, WCIT expressly excluded civil society organizations from participation and was held behind closed doors. This was widely considered a decisive feature of the summit, and the target of much public criticism online. In this regard, the prominence of hashtags like #WCITleaks, #leak, #anonymous, and #opwcit (for operation WCIT) on Twitter are not necessarily a sign that WCIT had been hijacked by generic online campaigns on this platform but may be interpreted in substantive terms. In other words, specifically digital interventions such as an online data dump cannot as a matter of course be considered “external” to controversy proper.

This discussion can also help us to articulate the problem with the precautionary approach to digital bias, which proposes to strip controversies of effects that are specific to the digital settings in which they are enacted. It is not in a good position to appreciate that media technological interventions (like a leak or the high volume of tweets that announced it) may present a significant contribution to public controversy. Precautionists wrongly suggest that the empirical object—controversies—should remain the same “with or without digital media,” as if their form, content, and character is and/or should be unaffected by the media technological settings in which they unfold. However, “inform-actional” formats—like leaks, or social media “trends,” and so on—may well influence the very form that public controversies are taking in the context of digitization (Anderson and Kreiss 2013). While informed by important methodological concerns with bias, the precautonists’ endeavor to “dis-embed” controversies from digital media settings could result in distortions of the empirical object.

This is not the place to discuss the digital transformation of forms of publicity in detail, but there is one development that I would like to mention here, because it is likely to affect the role and status of public controversy in digital societies, that is, the changing role and status of “issue dynamics” in informational environments.^14^ As has been discussed extensively by digital media scholars, digital platforms and infrastructures are increasingly oriented toward the dynamic valorization of content: search engines privilege fresh information, and social media seek to keep their users engaged by continuously informing them of “what is happening” (Gillespie 2013; Rogers 2013; see also Marres and Weltevrede 2013). As a consequence, the formatting of topics as “happening issues” has become increasingly common as a way of promoting the visibility of topics in media environments. This in turn raises the question of whether the very distinction between stable and “active” topics of knowledge and interest is shifting today. Could it be that the digitization of public media and interaction is precipitating a *generalization* of issue dynamics? It can seem that today anything, from a toothbrush to the sighting of a strange species of dog, may become the focus of issue-making activity.

I can offer no more than a speculative hypothesis here, but these observations suggest that it would be unwise for digital controversy analysts to assume the stability of “public controversy” as an empirical object. If digital media technologies are leaving their traces in the very form, content, and character of public controversy, then this would surely present an important topic of inquiry for controversy analysis. We should then actively investigate in what forms, shapes, and genres public controversies arise in digital settings—not just to secure a viable methodological strategy but as part of the empirical project of controversy analysis. The investigation of how digital settings influence the public articulation of contested affairs must then become part of our empirical inquiry. Digital controversy analysts should ask not just substantive questions but also formal ones like how is doing issues through data leaks different from doing issues with press releases?^15^

If digital devices play a role in the organization of public controversy, then controversy may be constituted differently depending on what devices and formats are deployed in its enactment. Indeed, it is now no longer self-evident why we would privilege public controversy as the focus of empirical analysis, because public engagement with contested affairs may also take other forms. Thus, in our analysis of the WCIT hashtags, hashtags associated with corporate advocacy (#freeandopen), hactivist campaigning (#opwcit), and small talk (#justsaying) turned out to be prominent alongside more issue-specific hashtags (#humanrights and #dpi for “deep packet inspection”). If we adopted a precautionary approach in digital controversy analysis, we could be tempted to disregard the former hashtags as a distraction from the WCIT controversy proper, that is, the substantive issues. But their prominence on Twitter can also be taken to suggest that a variety of different types of issue engagements were facilitated by this platform, from informal conversation to corporate advocacy and hactivist intervention, and that these types of engagements in particular gained prominence in relation to WCIT in this setting. When we analyze controversial issues with online media technologies, the form of controversy emerges as a relevant empirical question, that is, does WCIT primarily feature as an object of activist mobilization or a topic of expert disagreement, or a combination thereof? Controversy may have to be regarded as one format of issue articulation among others.

This has implications for our framing of the empirical object of digital controversy analysis. If we are serious about affirming the role of digital settings in controversy, then we should adopt a more open-ended empirical approach and map issues, not just controversies.^16^ To propose this is to further elaborate the empiricist commitment of controversy analysis. Classic work in STS has famously posited that controversies are analytically useful for social inquiry, insofar as these events render available wider social relations for empirical analysis. In turning to digital settings to analyze controversies, however, a different set of questions arises. As noted, issue mapping online shifts the emphasis to issue detection. We ask, is this topic really an active issue? One of the classic innovations of controversy analysis as an STS method was to defer to the empirical setting in answering substantive questions like Who are the protagonists? What is the topic of contention (Latour 2005)? In doing controversy analysis with digital platforms, we defer a further question to the empirical, that is, what form does engagement with the issue take? Are they topics of public debate or objects of activist mobilization? Are they thematized through information leaks or through the promotion of factual statements? The analytic sequence of digital controversy analysis is different. Whereas controversy analysis used to begin with a robust controversy in order to detect given actor relations, issue mapping begins with a given topic in order to detect emerging issue formations.^17^

To be clear, while the move from controversy analysis to issue mapping is informed by an affirmative understanding of digital bias, it is certainly *not* an uncritical approach. That controversies in digital settings so often revolve around “campaigns,” “gaffes,” and “publicity initiatives” is surely a problematic development. Not unrelatedly, some commentators now talk about digital “issue fatigue” (Oliver Burkeman’s blog 2013). Digitization doesn’t seem to favor the type of issue dynamics that historically have been appreciated by controversy analysts, that is, those that involve the articulation of clear points of contention, effectively address institutional actors, and have the capacity to produce enduring shifts in actor alliances and the balance of power.^18^ However, precisely because of their unsettling effects on public controversy, the emergence of digital forms of publicity requires our empirical attention. It is with this critical aim in mind that I propose to expand the scope of inquiry from controversy to issues. As is clear by now, this creates a significant degree of uncertainty about our empirical object. To conclude this article, I would like to show that digital methods of issue mapping can also be used to reduce this uncertainty.

## Mapping Issues with, and Against, Digital Media Technologies

Informational (or “inform-actional”) dynamics like linking and tagging may be indicative of issue formation, but these digital practices are nevertheless biased toward highly particular dynamics, not least the promotional effects of hyping and trending. This makes it necessary to take steps to ensure that issue mapping research actually maps issues. On the one hand, it is crucial that we accept the inherent ambiguity of the empirical object—issue formation involves both substantive and media technological dynamics. On the other hand, issue mapping should actively mitigate against the collapse of the former into the latter, whereby issue formation would be reducible to media technological processes. We must then treat the ambiguity of online issue formations as a topic of *critical* inquiry. Issue mapping research should not assume the platform’s definition of what counts as a relevant issue when we derive our indicators of issue activity from specifically digital formats—like hashtags or edits.^19^ From the standpoint of Twitter and Wikipedia, a topic becomes an issue when tagging and editing activity in relation to a topic intensifies, when the issue appears in Twitter’s list of “top trends” or Wikipedia’s “list of controversies” (http://en.wikipedia.org/wiki/List_of_Wikipedia_controversies; accessed December 2014). However, it is far from self-evident that the intensification of editing or tagging activity is the relevant criterion of issue formation from the standpoint of political epistemology. It won’t do for issue mapping research to call an “issue” whatever the platform says is one.

The inherent ambiguity of issue formations online then also works the other way, that is, for a topic to count as an issue, it must be *collectively* accomplished as such by the various actors and entities involved. As such, it cannot be reducible to digital settings and dynamics. If we are to advance the purposes of issue mapping as a social research approach, we must then do more than “follow the media” (Rogers 2009). We must push back against digital settings in equal measure by putting safeguards in place to ensure that our analysis reveals issue-specific activity and not just medium-specific features of the formations under study. We must prevent online issue analysis from uncritically going along with digital platform settings in their operationalization of what counts as an issue. A last example from our WCIT pilot study can help to clarify what such a critical but affirmative approach to digital issue mapping would entail.

**Figure 3.**
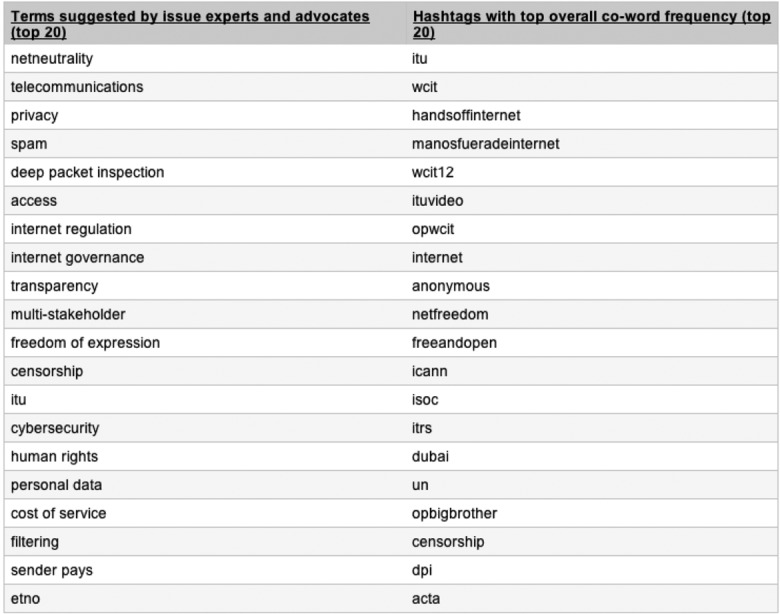
World Conference on International Telecommunications (WCIT) issue terms suggested by respondents and by Twitter, December 2012.

We realized at an early stage that by relying on hashtag analysis to qualify the issues of WCIT, our study was at risk of being overdetermined by Twitter, and we devised a number of ways to militate against this form of platform bias. We used a form of hashtag analysis that would minimize the influence of the promotional dynamics of Twitter by analyzing not how often hashtags occur (a frequence-based measure), but rather the relations between them, detecting which hashtags occur together in Tweets (a co-occurrence measure). This helped to militate against sudden bursts of key word occurrence, which tend to derive purely from massive re-tweeting and related efforts to get a hashtag to “trend” on Twitter (for a more detailed discussion of co-occurrence methods, see Marres and Gerlitz in press). Second, to determine which issue terms to map with Twitter, we did not just rely on the platform itself but also consulted issue experts and activists working in the area of Internet governance.^20^ Intriguingly, the issues identified by advocates were very different from those that our hashtag analysis identified as relevant (i.e., well connected; see Figure 3). Many of the Twitter-derived terms referred to Internet-based campaigns, while the expert and advocates singled out substantive issues. From the start, it was clear that the “issues of the platform” couldn’t be conflated with the “issues of the field.”^21^

Finally, we actively involved the issue activists and experts in the project of interpreting our issue and hashtag profiles, by inviting them to comment on the WCIT hashtag profiles published on our wiki site, Issue Mapping Online. Their responses differed widely, that is, some provided constructive commentary, for instance, by pointing us to the sites where #WCITleaks occurred and were discussed. Others were critical of our Twitter analysis. One respondent noted, “Having been on the ground in Dubai, involved in substantial planning w/NGOs leading up to the event over many months, and participating in the US delegation (and conversations w/many other governments’ officials), I must tell you I don’t see much connection between this analysis and what actually happened” (e-mail response received April 17, 2013). We found this especially interesting, because it compared what happened “on the ground” to Twitter and Twitter analysis and thereby inadvertently underlined the rift between civic engagement with WCIT in digital settings and the conference proceedings #behindcloseddoors. It helped to convince us that the famous opposition between the online and the off-line does not just present a methodological problem. Rather the question of which settings qualify as relevant locations for issue formation was clearly at stake in the controversy and featured as an issue in and of itself.

Our study of WCIT with Twitter is discussed in more detail elsewhere, (for a more detailed account, see http://issuemapping.net/Main/WCITProfiles; accessed December 2014) but this brief account should make clear that online analysis can be configured to ensure that it serves the substantive ends of issue mapping. In analyzing issues with digital settings, we can and must take specific steps to resist the capture of our analysis by media technological dynamics, such as those of Twitter in relation to WCIT, where campaign key words were pushed to the top of rankings by massive, often automated (re-)tweeting offensives in the effort to make particular terms trend. However, I have argued that in militating against platform bias, it should not be our objective to remove the traces of digital devices from our data in order to offer a “neutral” presentation of controversy. Rather, we should specify how digital settings participate in issue formation, alongside and in close association with an open-ended set of other equally partial entities. In doing so, our overall objective should be to *qualify* issue formation, not in the restricted, anti-quantitative sense of determining their “meaning,” but in the broad sense of establishing what forms of intervention are enabled in the process of issue articulation.

## Conclusion

One of the critical questions facing controversy analysis today is how it positions itself in relation to prominent digital platforms and infrastructures, such as search engines and social media. Will controversy analysis as a digital method align itself with the methods, features, and objectives promoted by and through prominent platforms? Or will controversy analysis take the form of *a reconstructive project*, one that actively configures a digital apparatus to serve the empirical ends of issue analysis? The latter requires that we recognize that controversy analysis is *always partial*, and that it is our task to formulate a methodological strategy that is partial to the intellectual and normative aims of the study of STS. My argument may seem paradoxical but is not. If we want to ensure that controversy analysis as a digital method enables substantive research on issue formation, then we must not seek to bracket the role of digital technology in controversy, but instead closely engage with the phenomenon of “digital bias,” and offer an affirmative but critical assessment of how the digital participates in controversy and issue formation.

Of the three frameworks discussed above—demarcation, discourse analysis, and radical empiricism—the last is best equipped to realize this objective. Demarcationist and discursivist approaches to controversy analysis are also centrally concerned with problems of digital bias, and they too configure controversy analysis as a way to address these very problems. However, these approaches tend to define the “influence of digital settings” in negative terms and presume that to analyze controversies with digital methods, we must *bracket* the influence of digital settings on controversy, that is, digital bias undermines the substantive concerns of controversy analysis. As such, they leave unchallenged our blind spots with respect to the participation of media technologies in controversy and are unable to address a central question of how digital media technologies participate in the enactment of controversy.^22^ The problem with demarcationist and discursivist approaches is thus not the substantive aim of their projects—to adjudicate between sources, or to explore controversies—but the fact that they assume these projects require us to pay as little substantive attention as possible to digital technology itself.

Rather than treating digital bias as a negative phenomenon to be bracketed, we should then develop methodological and empirical tactics that address the question of how digital devices participate in the enactment of controversy and the formation of issues. Such an approach is not without risks, and it has consequences for the very framing of controversy analysis. Once we affirm that media technologies always participate in the enactment and analysis of controversies by digital means, then we must broaden the empirical focus of controversy research, that is, we should not only analyze controversies but also map issues. That is to say, we should not limit our analysis to topics that are subject to explicit and focused disagreement among actors but equally investigate a broader range of engagements with public affairs that may be indicative of media technological “takeover” of the process of issue formation or actually enable substantive engagement.

The move from controversy analysis to issue mapping entails a significant shift in empirical focus and *extends* two long-standing commitments of controversy analysis as an STS method. Turning to digital settings to analyze controversies, these settings become empirical resources that allow us to address questions like, is this topic an issue? where is it happening, and what forms does it take? It allows us to move beyond impartiality in the study of science, technology and society by digital means. Controversy analysis came to play a pivotal role in the development of STS precisely *because* it enabled the operationalization of this intellectual project. The shift from controversy analysis to issue mapping in digital research extends this “move beyond impartiality.” It takes up the affirmative argument that all knowledge contents are marked by bias, and extends it to the media technological settings of public life. All sites of publicity come with biases. They pose important problems both for the conduct of public controversy and for controversy analysis, and they deserve to be investigated rather than bracketed.

As I have shown, there are important precedents in the STS literature for such a proposal, and the digital implementation of controversy analysis offers significant opportunities to further develop the reflexive and experimental methodological sensibilities for which the field is well known. Faced with the significant biases that digital media technologies introduce in the enactment and analysis of controversy, it might be tempting to some to look for safety in the semblance of neutrality offered by established empirical methodology. In my view, we should actively resist the temptation to reach for ideals of epistemic impartiality, which STS has so convincingly shown to be flawed. This field offers significant conceptual and methodological resources for the development of a *partial methodology* for researching controversy by digital means, one that suspends the ideal of the neutrality of digital settings without however sacrificing the substantive focus of digital research on issue formation.

## Notes

1.
Morozov (2012) proposes that “whenever users are presented with search results that are likely to send them to sites run by pseudoscientists or conspiracy theorists, Google may simply display a huge red banner asking users to exercise caution and check a previously generated list of authoritative resources before making up their minds.”

2. Controversy analysis can also be called inherently interdisciplinary insofar as it relies on a diverse set of competences, that is, social research, computing, graphic design, and so on.

3. This prototype application was developed by Intel’s Confrontational Computing Program. Implemented as a browser extension, the tool “highlights disputes on the web” with a pop-up window presenting an overview of “evidence pro- and con-” (Ennals et al. 2010). Development was stopped in 2011.

4. In the context of digitally induced “information overload,” knowledge controversy is appreciated not just as a marker of legitimacy but also of relevance: that which is currently contested on substantive grounds deserves our attention.

5. In other cases, a causal explanatory framework is adopted, as when researchers aim to establish the relative influence of a particular actor grouping, type of argument, or form of public intervention, in an effort to demonstrate who or what “decided” the issue (Benkler 2012). Such a causal approach is in tension with the insights about coproduction and the heterogeneous composition of action as championed in science, technology, and society (STS).

6. Besides actor–network theory, scientometrics has been an important influence on the development of controversy analysis as a digital method (Scharnhorst and Wouters 2006), and in what follows I will explore the connections between these traditions.

7. Such STS-inspired approaches can be called “object centered” insofar as they propose that the formation and transformation of issues itself is the primary dynamic to be detected and analyzed. This approach differs from actor-centered approaches, in which the mobilization of organizations, individuals, and institutions figures as the primary dynamic, something which is then expected to account for the substantive framing and reframing of issues (see Chateauraynaud 2009).

8. The problem of digital bias is particularly complex, as such bias derives from multiple devices, from search engines to browsers, application program interfaces, and so on, as well as from the instruments of controversy analysis themselves. Furthermore, digital bias is not limited to online settings but also affects off-line data sets as, for example, a corpus of policy reports contained in a digital database. Online settings nevertheless present an especially clear case of digital bias, and much data today are marked by their mediation in these settings.

9. This problem of the “influence of the setting” (Garfinkel 1967) and efforts to contain it by dis-embedding empirical phenomena from the settings in which they occur are not just relevant to controversy analysis but also to a wide range of social research methodologies, including survey methods (Savage 2012) and content analysis (Herring 2010). My account in this article is limited to controversy analysis. I argue that controversy analysis as an STS method offers special resources for dealing with the intractable problem of digital bias.

10. There are many other differences: precautionists tend to work with stable data sets, while many of those who affirm the bias of the setting are attracted by the dynamic data sets that online platforms make available.

11. This affirmative use of citations has not prevented citation analysts from criticizing the biases that citations and indexing devices introduce into the scientific literature. For instance, they identified the problematic that well-cited sources attract more citations for the simple reason that they are well cited. Indeed, it was to militate against these very biases that some of the important methodological innovations in citation analysis—such as cocitation analysis—were developed.

12. This hashtag profile was produced with the aid of the associational profiler, a tool in progress developed by me and colleagues at Goldsmiths and the University of Amsterdam, in order to analyze issue activity with digital methods. The profiler applies co-occurrence measures in order to detect associations between key words, and plots relations in these key word relations over time, from interval to interval. The colors indicate high (blue) and low (red) specificity, that is whether terms appear with one another proportionally more often than with other terms (hashtags) in the data set. The data set for our WCIT study included all tweets that contain the words WCIT or ITU (for UN International Telecommunications Union, which hosted the conference), posted between November 23, 2012, and December 19, 2012 inclusive, the period within which the two-week summit took place, which we divided into four intervals of about a week. Our data set contained 108,781 tweets. This WCIT study was designed to trial the associational profiler tool. For more information, see http://issuemapping.net/Main/WCITProfiles (accessed December 2014).

13. It is probably for this reason that some STS-informed work advocates a platform-independent approach to digital controversy analysis (Venturini and Guide 2013; Beck and Kropp 2011).

14. To make matters more complicated, this is a reflexive effect. The dynamization of digital content is partly a consequence of the implementation of methods of network and textual analysis in digital infrastructures. Platforms like Google and Twitter increasingly rely on such methods of data analytics to valuate, select, and push content. I discuss the implications of these reflexive effects for the politics of STS methods elsewhere (Marres and Gerlitz in press).

15. The role of issue framing has been of long-standing interest in policy analysis, and as the digital implementation of controversy analysis raises the question of the information and action format, controversy analysis may significantly benefit from exchanges with this field. Here, however, I am primarily interested in the methodological framing of controversy analysis as a partial methodology informed by STS.

16. The focus on “public controversy” in the study of knowledge politics in STS has been criticized before, among others by Annemarie Mol (2002) who suggested that to analyze controversies is to privilege the evolution of arguments over time and entails a disregard for situated practices in which problems make themselves felt. But while Mol’s critique mobilized ethnography against scientometrics, this article offers a mixed methodology.

17. If we inflate this distinction, it begins to resemble the difference between studying social order and researching social change. In issue mapping, the aim is to determine which topics become the scene of socio-tech-epistemic-and-so-on transformation. In controversy analysis, however, the aim has often been to trace the relations between actors that constitute the taken-for-granted background of social life, that is, its “order.”

18. Arguably, controversy analysts in STS as in other fields have privileged sustained forms of issue engagement facilitated by peer-reviewed publications, public consultation events, and public policy debate. Digital platforms like Twitter facilitate engagement with similar topics (climate change, Internet governance) but the style and tone is often humorous, and timeliness and “controversy are frequently short-lived.

19. For a discussion of the inherent partiality of theWikipedia platform, see Tkacz (2014).

20. In the week before the conference started, we sent out an e-mail survey to roughly twenty-five issue advocates and experts active in the area of Internet governance, asking them to name five issues they considered especially relevant to WCIT.

21. Of the top twenty issue terms on the lists of Twitter terms and expert terms, only two were the same.

22. Where proponents of these approaches do rely on platform-specific formats, such as Wikipedia edits, they tend to frame this reliance in purely instrumental terms and remain silent about the active role played by these devices in the formatting of “controversy” itself.
